# Moving lupus epidemiology forward: returning to the basics

**DOI:** 10.1186/ar4617

**Published:** 2014-09-18

**Authors:** Julia F Simard

**Affiliations:** 1 Stanford School of Medicine, Stanford, CA, USA

## 

The protracted diagnostic period and variable disease presentation not only complicate diagnosing SLE but also the epidemiologic study of it. Coupled with the remitting and relapsing nature of the disease and the challenges in managing it, clinical research in lupus requires careful attention to study design, control selection, temporality, and many often overlooked issues in the analysis phase. Between "big data" and the impressive advances in the basic sciences, it is tempting to either oversimplify methods to take advantage of "big data" or overcomplicate because the problem itself is complicated.

As we revisit the building blocks of epidemiologic research, we will uncover opportunities to move epidemiology and clinical research forward in SLE. Why do we care about effect modification and what is it? Why can we not just adjust for everything that we want to? And perhaps, most importantly, going back to the very beginning and asking ourselves: does this matter?

During this talk we will discuss issues relating to case identification methods, potential biases associated with control selection, and return to the basics of epidemiologic research. Although we shall discuss these issues in the context of environmental (nongenetic) factors, these concerns extend across the worlds of observational data analysis, can impact randomized trials, and are relevant for all types of exposures and outcomes.

